# A Network Meta-Analysis of the Relative Efficacy of Treatments for Actinic Keratosis of the Face or Scalp in Europe

**DOI:** 10.1371/journal.pone.0096829

**Published:** 2014-06-03

**Authors:** Stefan Vegter, Keith Tolley

**Affiliations:** 1 University of Groningen, Department of Pharmacy, Groningen, the Netherlands; 2 Vegter Health Economic Research, Groningen, the Netherlands; 3 Tolley Health Economics Consultancy Ltd, Buxton, United Kingdom; Groningen Research Institute of Pharmacy, Netherlands

## Abstract

**Background:**

Several treatments are available for actinic keratosis (AK) on the face and scalp. Most treatment modalities were compared to placebo and therefore little is known on their relative efficacy.

**Objectives:**

To compare the different treatments for mild to moderate AK on the face and scalp available in clinical practice in Europe.

**Methods:**

A network meta-analysis (NMA) was performed on the outcome “complete patient clearance”. Ten treatment modalities were included: two 5-aminolaevulinic acid photodynamic therapies (ALA-PDT), applied as gel (BF-200 ALA) or patch; methyl-aminolevulinate photodynamic therapy (MAL-PDT); three modalities with imiquimod (IMI), applied as a 4-week or 16-week course with 5% imiquimod, or a 2–3 week course with 3.75% imiquimod; cryotherapy; diclofenac 3% in 2.5% hyaluronic acid; 0.5% 5-fluorouracil (5-FU); and ingenol mebutate (IMB). The only data available for 5% 5-FU was from one small study and was determined to be too limited to be reliably included in the analysis. For BF-200 ALA and MAL-PDT, data from illumination with narrow-band lights were selected as these are typically used in clinical practice. The NMA was performed with a random-effects Bayesian model.

**Results:**

25 trials on 5,562 patients were included in the NMA. All active treatments were significantly better than placebo. BF-200 ALA showed the highest efficacy compared to placebo to achieve total patient clearance. BF-200 ALA had the highest probability to be the best treatment and the highest SUCRA score (64.8% and 92.1%), followed by IMI 5% 4 weeks (10.1% and 74.2%) and 5-FU 0.5% (7.2% and 66.8%).

**Conclusions:**

This NMA showed that BF-200 ALA, using narrow-band lights, was the most efficacious treatment for mild to moderate AK on the face and scalp. This analysis is relevant for clinical decision making and health technology assessment, assisting the improved management of AK.

## Introduction

Actinic keratosis (AK) is a premalignant skin condition, characterised by thick, scaly, or crusty patches on the skin. The lesions can be located on the face, ears, neck, scalp, chest, hands, forearms, or lips. A common synonym of actinic keratosis is solar keratosis, as it is predominantly caused by prolonged and unprotected exposure to sunlight. Male gender, older age, light pigmentation status (Fitzpatrick skin types I and II), baldness, skin wrinkling, and extensive history for sunburn are risk factors for AK [1]. AK is considered as a pre-cancerous condition, since there is a continuous annual risk of lesions progressing to squamous cell carcinoma (SCC). In various epidemiological studies the risk for progression from AK to SCC has been estimated between nil and 0.53% per lesion per year [2]–[4]. Over 10 to 25 years, the estimated progression from AK to SCC has been estimated between 5% and 20% [5]. AK is one of the most common conditions treated by dermatologists (third most common reason for consulting a dermatologist [6]) and progression to SCC can impact on patient health related quality of life (HRQoL) [7]. The primary goal of AK treatment is to achieve complete clearance of lesions, thereby eliminating the risk of progression to SCC. The removal of visible lesions may additionally improve patient HRQoL [8], [9]. Available AK treatments used in clinical practice include topical treatments (such as diclofenac (DCF); 5-fluorouracil (5-FU), imiquimod (IMI), ingenol mebutate (IMB)), cryotherapy, and photodynamic therapy (PDT) using alternative photosensitizing agents including 5-aminolaevulinic acid (ALA) or methyl aminolevulinate (MAL).

When comparing the effectiveness of two or more interventions, randomized clinical trials (RCTs) that compare the interventions directly (head-to-head trials) are often preferred for health technology assessment and reimbursement decision making. In AK, most but not all published trials are placebo-controlled studies, limiting the potential to compare active treatments. Network meta-analyses (NMA), can provide a valid statistical estimate of the comparative efficacy of different treatment modalities by combining in a network of evidence both direct head-to-head and indirect comparative evidence [10]–[13]. A NMA of different treatments in AK has recently been published [14]. This study was performed as part of a Cochrane Review [15]. The NMA however grouped all the ALA-PDT and different imiquimod (IMI) treatments. Therefore, to increase the value for clinical and reimbursement decision making purposes the objective of this study was to perform a Bayesian NMA in order to provide the most up to date assessment of the comparative efficacy of available treatment modalities for mild to moderate AK on the face or scalp, including different treatment modalities with ALA-PDT and imiquimod. The NMA will be performed from a European perspective, thereby focusing on treatments available and regularly used in clinical practice in Europe. The starting point for our NMA was to utilise the studies identified in the recent Cochrane Review of AK treatments [15]. Results from this NMA may be used as the source of clinical efficacy data in economic evaluations of the cost-effectiveness of AK treatments.

## Methods

### Study selection

The recent Cochrane systematic search and review was used to identify studies on treatments for AK, provide information on literature search strategies and on the risk of bias for the included studies.^15^ In the Cochrane review, databases were searched up to March 2011; a final prepublication search was performed in April 2012 but these were not described in the Cochrane review (these studies were listed as ‘awaiting classification’). The Cochrane review included 83 studies; 12 studies were listed as ‘awaiting classification’; and several on-going studies were identified. The studies included in the Cochrane review and the studies awaiting classification were assessed for inclusion in the NMA; furthermore, the status of the ongoing studies was also reassessed in January 2014. No new systematic literature review was performed, but extensive literature searches by the authors did not result in new trials being identified. Two dermatology consultants reviewed the inclusion and exclusion criteria for the clinical trials and the design of the NMA (see Acknowledgement section). Both authors checked the studies identified in the Cochrane NMA for inclusion in our updated NMA, in order to ensure the studies included were comparable in terms of study design, treatment modality studied and patient characteristics. Any disagreements regarding study selection were resolved by consensus. Both published and unpublished randomized controlled trials (RCT's) were considered for inclusion. RCT's using intra-individual designs (e.g. treatments applied to opposite sides of the face) were excluded. Studies needed to report intention to treat (ITT) or full analysis set (FAS) data, not only per-protocol (PP) data. Evaluation of efficacy needed to be a minimum of one month after the end of treatment, (EOT) but no more than 1 year post-treatment.

### Types of participants and treatments

Studies on participants with mild to moderate AK on the face or scalp were included, defined as having between 5 and 20 lesions. Studies with immunosuppressed participants were excluded. Studies on combination therapies were excluded, as the focus of the NMA was on the efficacy of the individual treatment options. Trials studying dose variations of a single treatment (e.g. dose-ranging studies) or unconventional treatment dosages or schedules (e.g. 3- or 8-week courses of IMI 5%) were also excluded as these could not be included in a treatment network. However, small differences in treatment dosages or schedules were considered to be equivalent, such as IMI 5% applied 2 or 3 times per week. The present analysis was performed from a European clinical practice perspective; trials on ALA stick [16] were excluded as this treatment is not available in Europe. However, this treatment was included in a scenario analysis enabling the assessment of its relative efficacy. For trials with BF-200 ALA and MAL-PDT, efficacy data using narrow-band light sources (LED lights) were used [17]–[20], as narrow-band lights are the standard light source typically used in clinical practice.

Eleven treatment modalities were included in the NMA: three modalities with 5-aminolaevulinic acid (ALA)-photodynamic therapy (PDT), applied as a gel or patch; methyl aminolaevulinate (MAL)-PDT; three modalities with imiquimod (IMI), applied as a 4-week course with 5% IMI, a 16-week course of 5% IMI or a 2–3 week course with IMI 3.75%; cryotherapy; diclofenac 3% in 2.5% hyaluronic acid (DCF); 0.5% 5-fluorouracil (5-FU); and ingenol mebutate (IMB). All vehicle and placebo treatment arms, including placebo-PDT, were considered to be equivalent and were treated as a single arm in the NMA. Treatment with 5% 5-fluorouracil (5-FU) was also considered. However, because the only included study [21] with this treatment was very small and reported a clearance rate (23 out of 24 patients) that was not consistent with the literature [22], this treatment was not included in the NMA.

### Outcome measures

In line with the Cochrane review, the primary read-out considered was ‘complete patient clearance’, i.e. total clearance of all of a patient's lesions. Studies that reported only other outcomes, such as number of lesions cleared or partial participant clearance, were not included. For treatments that allowed for multiple treatment courses, including MAL-PDT, BF-200 ALA and IMI 5% 4-week course, the clearance rates after the (optional) second course were used in the NMA. Both target-lesion and all-lesion studies were included but for studies presenting both outcomes only the all-lesions outcome was used in the NMA.

### Network meta-analysis

A Bayesian random-effects NMA for multi-arm trials based on the model provided by the University of Bristol in the UK [23] was used to analyse the efficacy of all treatments in the network simultaneously. A fixed-effects model was considered but the model fit of the random-effects model was considerably better based on Deviance Information Criteria (DIC) and residual deviance statistics. Three chains with 10,000 iterations and a burn-in of 2000 iterations were run using non-informative priors. The main outcome parameter of the NMA was the probability to achieve total patient clearance, expressed in log OR relative to the other treatments or placebo. The patient clearance rates were also calculated for each treatment. The estimated treatment effect size and associated uncertainty was translated into the probability that a certain treatment was the ‘best’ (i.e. most effective treatment). An alternative ranking method, the surface under the cumulative ranking curve (SUCRA), was also calculated. SUCRA ranges from 0 to 1, where 1 reflects the best treatment with no uncertainty and 0 reflects the worst treatment with no uncertainty [24]. Inconsistency between direct and indirect evidence in the NMA was estimated as the weighted difference between the indirect and direct estimate for a randomly chosen contrast, using the package ‘MTcoherence.fun’ [25]. The program Winbugs 1.4 statistical software (MRC Biostatistics Unit, Cambridge, UK) was used for the NMA.

In addition to the NMA, simple weighted averages for complete patient clearance were calculated per treatment arm; (i.e. the number of patients with complete patient clearance divided by the total number of patients) with 95% confidence intervals calculated by assuming binomial distributions. The results from this naïve meta-analysis were compared to the results from the NMA. Linear regression analyses were performed to test for associations between patient characteristics, reported on trial level, and patient complete clearance in the placebo arms of the included studies. Fixed-effects (FE) and random-effects (RE) direct meta-analyses were performed for all studies comparisons. Tests for heterogeneity were performed but as a default the results from the random-effects models were reported where available to allow for heterogeneity in the studies included. The program R 3.0.1 statistical software was used for the direct meta-analysis, using the package ‘rmeta.’

## Results

### Study inclusion

**Figure 1:**
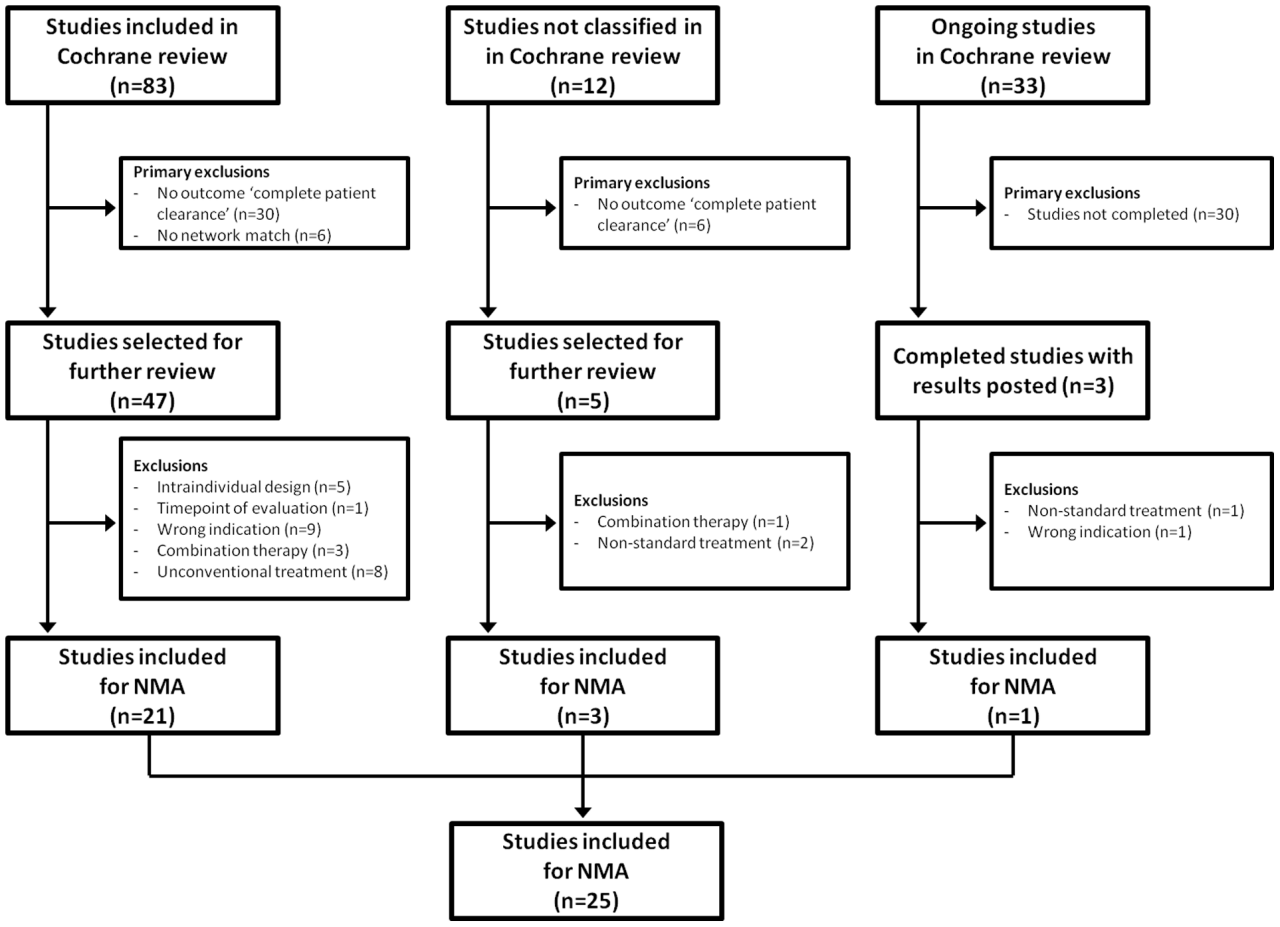
Flow-chart of study selection.

The studies included in the Cochrane review were independently assessed for suitability for inclusion in the NMA. An overview of the selection process is shown in Fig. 1. Reasons for study exclusion from the NMA were that they did not report the outcome ‘complete patient clearance’ (36 studies); did not fit in a treatment network (6 studies); were for the wrong indication (4 studies in immunocompromised patients; 6 studies not for mild to moderate AK on face or scalp); or for various other reasons such as an intra-individual study design or treatment with combination therapy. A detailed overview of study exclusion criteria is shown in Table S1. A total of 25 studies were available for inclusion in the NMA.

### Trial and patient characteristics

An overview of the included studies and patient characteristics is shown in Table 1. Most included studies were placebo-controlled studies, only 1 study compared only active treatments.^28^ The trials included a total of 5,562 AK patients. The average patient age in the studies ranged from 63.2 to 71.9 years; the majority (81.4%) were male. The average number of lesions per patients varied from 5.6 to 15.5. Olsen scores (AK lesion severity) and Fitzpatrick skin types were not reported in most studies (15 and 11 studies, respectively). The patient characteristics were similar between the interventions. In univariate or multivariate linear regression analyses, there were no significant associations between complete patient clearance and age, gender, number of lesions at baseline, Olsen score or Fitzpatrick skin type.

**Table 1:** Study and participant characteristics.

Author, year (ref)	Treatments	N	Age	Male	Lesions	Olsen score			Fitzpatrick skin type			
						I	II	III	I	II	III	IV–V
Szeimies, 2009 [18]	MAL-PDT; Placebo	115	68.2	79%	7.2	41%	59%	0%	19%	44%	27%	10%
Pariser, 2008 [17]	MAL-PDT; Placebo	96	66.4	82%	7.5	73%	27%	0%	23%	50%	27%	0%
Dirschka, 2012 [20]	BF-200 ALA; MAL PDT; Placebo	290	70.7	84%	6.2	39%	61%	0%	2%	35%	52%	12%
Szeimies, 2010 [19]	BF-200 ALA; Placebo	47	70.5	86%	5.6	54%	46%	0%	5%	58%	35%	2%
Hauschild, 2009a [31]	ALA-PDT patch; Placebo	99	70.7	82%	5.7	44%	56%	0%	9%	83%	9%	0%
Hauschild, 2009b [31]	ALA-PDT patch; Cryotherapy; Placebo	331	70.5	72%	5.6	44%	56%	0%	18%	66%	15%	1%
Szeimies, 2004 [40]	IMI 5% 16-week; Placebo	286	71.0	87%	5.8							
Korman, 2005 [53]	IMI 5% 16-week; Placebo	492	66.3	88%	4–8†							
NCT00828568* [54]	IMI 5% 16-week; Placebo	422	67.0	82%	4–8†							
Lebwohl, 2004 [38]	IMI 5% 4-week; Placebo	436	66.0	87%	6.0							
Jorizzo, 2007 [55]	IMI 5% 4-week; Placebo	246			6.0							
Alomar, 2007 [56]	IMI 5% 4-week; Placebo	259	71.1	88%	6.6				16%	50%	29%	5%
Krawtchenko,2007 [21]	IMI 5% 4-week; Cryotherapy	51	71.1	81%	8.0				19%	37%	43%	1%
Weiss, 2002 [57]	5-FU 0.5%; Placebo	98	63.2	87%	15.5				48%	52%	0%	0%
Jorizzo, 2002 [58]	5-FU 0.5%; Placebo	114		80%	15.0				86%	14%	0%	0%
Stockfleth, 2011 [29]	DCF; 5-FU 0.5%; Placebo	451	71.9	85%	5.7	40%	60%	0%				
Wolf, 2001 [59]	DCF; Placebo	117			≥5†							
Rivers, 2002 [60]	DCF; Placebo	97	67.5	76%	7.2	56%	39%	2%	16%	66%	18%	0%
Solaraze study 2 [61]	DCF; Placebo	108			≥5†							
Gebauer, 2003 [28]	DCF; Placebo	150	68.4	59%	10.6	29%	54%	17%				
Lebwohl, 2012 [39]	IMB; Placebo	547	65.1		4–8†							
NCT00700063 [62]	IMB; Placebo	65	68.3	86%	4–8†							
Swanson, 2010 [30]	IMI 3.75%; Placebo	319	64.4	82%	11.1				27%	27%	22%	24%
Hanke, 2010 [63]	IMI 3.75%; Placebo	326	64.0	79%	10.7				29%	29%	20%	22%

ALA: 5-aminolaevulinic acid; MAL: methyl aminolaevulinate; PDT: photodynamic therapy; IMI: imiquimod; DCF: diclofenac 3% in 2·5% hyaluronic acid (DCF); 5-FU: 5-fluorouracil; IMB: ingenol mebutate (IMB).

† average baseline lesions not reported in these studies, inclusion criteria shown.

* this study included two trials namely NCT00828568 Aldara and NCT00828568 Taro.

### Treatment network

**Figure 2:**
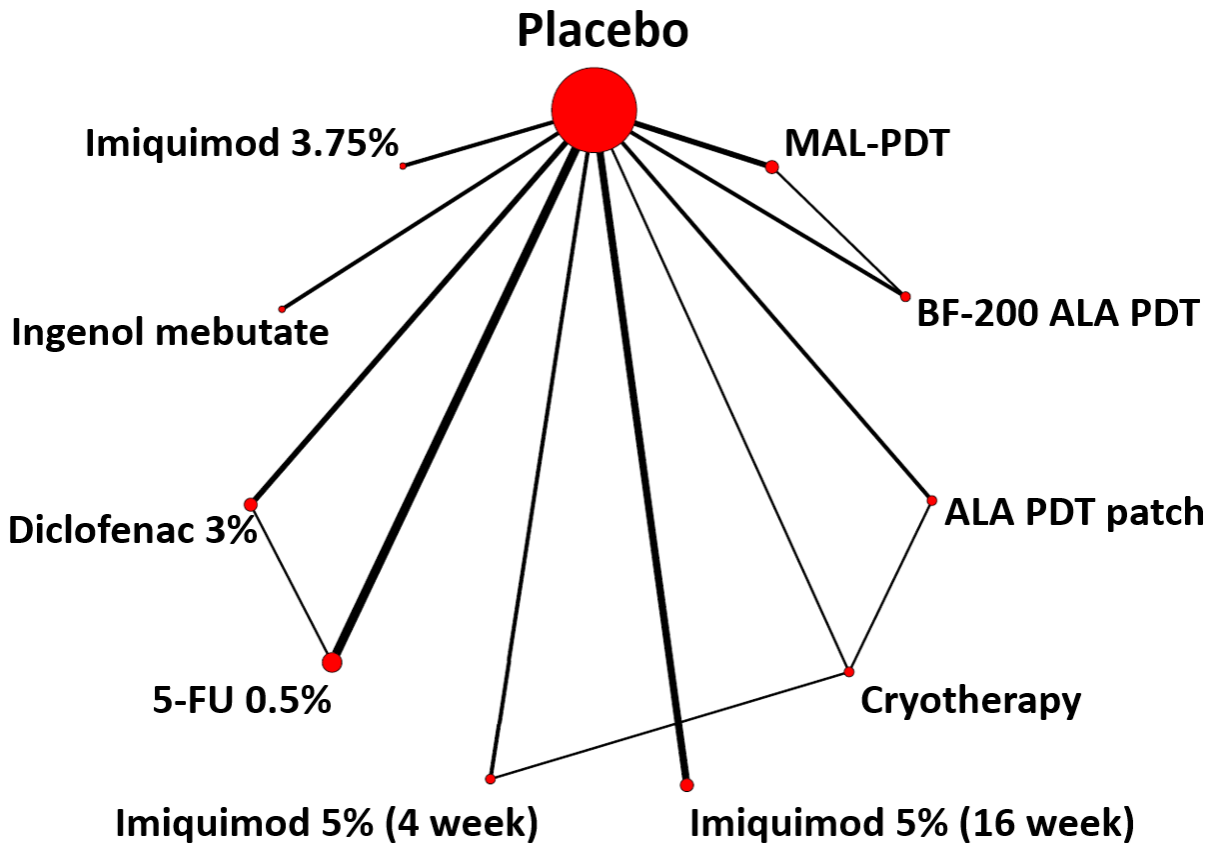
Treatment network for the NMA.

The treatment network for the NMA is shown in Fig. 2. Placebo treatment was a common reference comparator for all treatment arms but there were also several active treatment comparisons (direct head-to-head evidence) that could be included in the network. The number of patients treated with each therapy is shown in Table 2. Most patients were treated with Imiquimod (N = 1,566, with 966 of these receiving IMI 5% 16-weeks), followed by DCF (N = 413) and IMB (N = 309).

**Table 2:** Number of patients per treatment, naïve (averaged) clearance rates and clearance rates calculated with NMA.

	Number of studies	Number of patients	Clearance rate	
			Naïve meta-analysis*	NMA†
Placebo	23	2250	6.9% (5.9–8.0%)	6.9% (5.5–8.3%)
MAL-PDT	3	232	65.9% (59.9–72.0%)	54.8% (33.6–76.0%)
BF-200 ALA	2	156	85.3% (79.5–90.4%)	75.8% (55.4–96.2%)
ALA-PDT patch	2	205	62.0% (55.1–68.3%)	56.8% (30.5–83.1%)
Cryotherapy	2	169	49.1% (41.4–56.8%)	38.2% (12.1–64.3%)
Imiquimod 5% (16 weeks)	5	966	45.1% (42.0–48.2%)	63.3% (45.5–81.1%)
Imiquimod 5% (4 weeks)	3	278	57.2% (51.4–62.9%)	56.3% (33.8–78.8%)
Diclofenac 3%	5	413	35.4% (30.8–40.0%)	24.7% (12.4–37.0%)
5-FU 0.5%	3	262	54.6% (48.5–60.7%)	59.9% (38.9–80.9%)
Ingenol mebutate	2	309	43.0% (37.5–48.5%)	54.5% (27.8–81.2%)
Imiquimod 3.75% (4 weeks)	2	322	34.8% (29.5–40.1%)	39.9% (15.6–64.2%)

* calculated by dividing the number of patients with the outcome ‘complete patient clearance’ by the total number of patients for each treatment.

† calculated by applying the relative efficacies output from the NMA to the average clearance rate in the placebo treated patients.

ALA: 5-aminolaevulinic acid; MAL: methyl aminolaevulinate; PDT: photodynamic therapy; 5-FU: 5-Fluorouracil; NMA: Network meta-analysis.

### Network meta-analysis results

**Figure 3:**
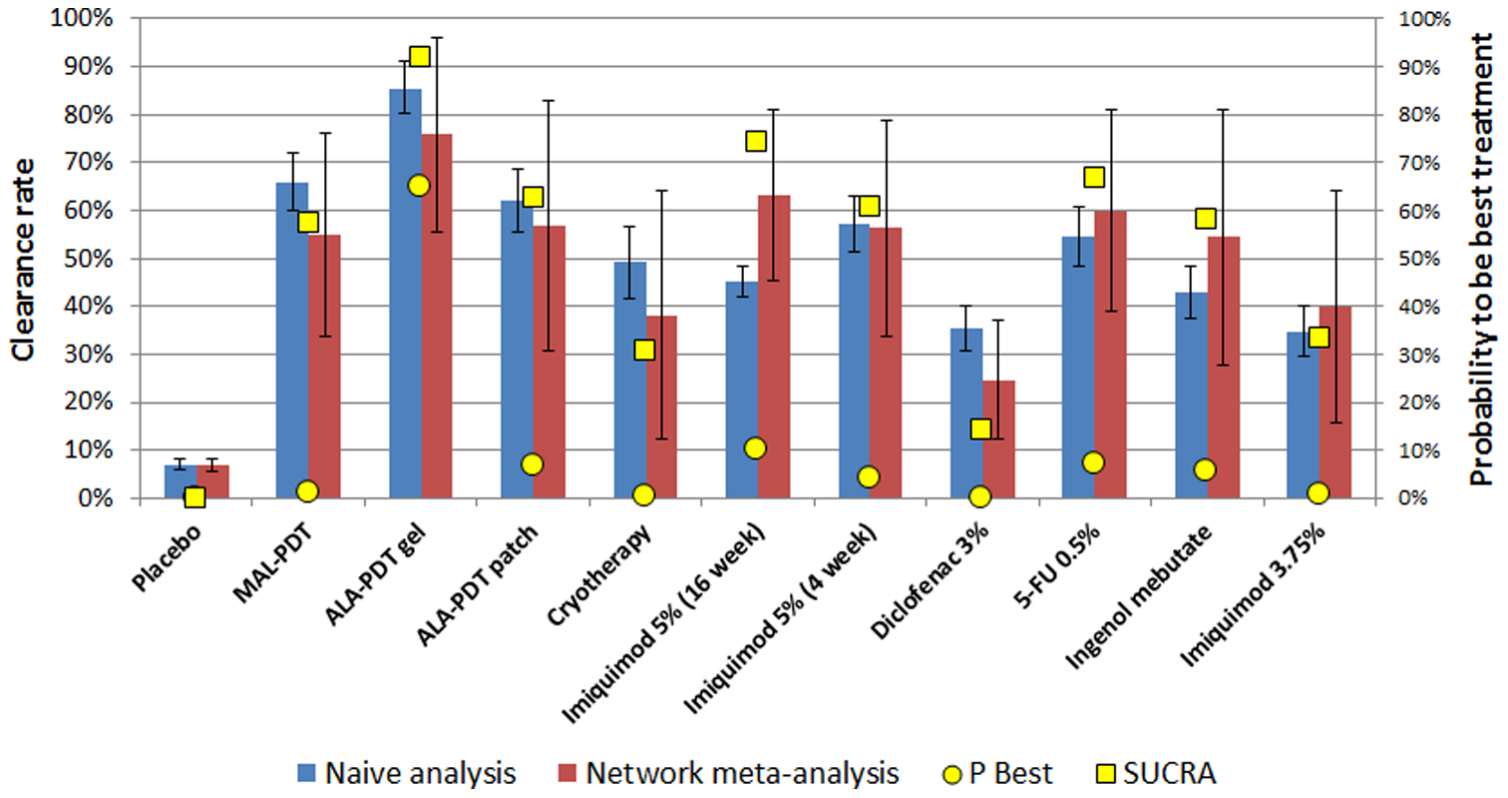
Absolute clearance rates (bars ± SE; left axis) and ranking according to the probability to be the best treatment (yellow dots; right axis) and the SUCRA score (yellow squares; right axis).

The NMA model converged and there were no significant inconsistencies between the direct and indirect evidence within the NMA. The estimated absolute clearance rates calculated from the NMA are shown in Table 2 and Figure 3. BF-200 ALA had the highest absolute complete clearance rate at 75.8% (95% CI: 55.4–96.2%), followed by 5-FU (59.9%, 95% CI: 38.9–80.9%), Imiquimod 16 weeks (63.3%, 95% CI: 45.5–81.1%), Imiquimod 4 weeks (56.3%, 95% CI: 33.8–78.8%) and ALA-PDT patch (56.8%, 95% CI: 30.5–82.1%) (Table 2). The findings were similar using a naïve meta-analysis approach, although there were some modest differences in absolute estimates (Table 2). Figure 3 shows the probabilities for each treatment to be the best (i.e. most effective) treatment in yellow dots while the SUCRA scores are shown in yellow squares. BF-200 ALA had the highest probability (64.8%) to be the most effective treatment, followed by Imiquimod 16 weeks (10.1%) and 5-FU 0.5% (7.2%). The ranking was similar when using SUCRA scores, being highest for BF-200 ALA (92.1%), followed by Imiquimod 16 weeks (74.2%) and 5-FU 0.5% (66.8%).

The results of the NMA and naïve meta-analysis in terms of Odds Ratios (OR) for complete clearance for each treatment vs. placebo are presented in Table 3. More extensive results for the relative efficacies of all included treatments is presented in Table 4. All active treatments in the analysis were significantly superior to placebo. BF-200 ALA was associated with an estimated OR of 45.9 (95% CI: 13.9–151.8), followed by IMI 5% 16-week, OR: 23.8 (10.4–54.2) and 5-FU 0.5%, OR: 20.7 (7.7–55.7).

**Table 3:** Efficacy of AK treatments for total patient clearance.

	OR for total patient clearance		Treatment ranking	
	Direct meta-analysis	Network meta-analysis	Probability to be best	SUCRA
Placebo	1 (reference)	1 (reference)	0.0%	0.0%
MAL-PDT	14.3 (7.1–28.6)	16.5 (6.5–42.1)	1.1%	57.2%
BF-200 ALA	40.1 (16.1–100.1)	45.9 (13.9–151.8)	64.8%	92.1%
ALA-PDT patch	16.7 (7.3–38.2)	18.1 (5.6–58.9)	6.7%	62.8%
Cryotherapy	7.3 (2.7–19.4)	8.0 (2.4–26.9)	0.3%	30.6%
Imiquimod 5% (16 weeks)	21.7 (10.9–42.9)	23.8 (10.4–54.2)	10.1%	74.2%
Imiquimod 5% (4 weeks)	17.5 (2.4–128.3)	17.6 (6.5–47.6)	3.9%	60.9%
Diclofenac 3%	3.4 (2.3–4.9)	4.3 (2.1–8.6)	0.0%	14.0%
5-FU 0.5%	20.5 (4.2–100.5)	20.7 (7.7–55.7)	7.2%	66.8%
Ingenol mebutate	16.8 (9.2–30.8)	16.4 (5.0–53.6)	5.5%	58.1%
Imiquimod 3.75% (4 weeks)	8.5 (5.1–14.3)	8.7 (2.9–26.2)	0.6%	33.2%

**Table 4:** Efficacy of AK treatments for total patient clearance; all treatments relative to each other.

	Placebo	MAL-PDT	ALA-PDT gel	ALA-PDT patch	Cryotherapy	Imiquimod 5% (16 week)	Imiquimod 5% (4 week)	Diclofenac 3%	5-FU	Ingenol mebutate	Imiquimod 3.75%
**Placebo**		**16.5 (6.5–42.1)**	**45.9 (13.9–151.8)**	**18.1 (5.6–58.9)**	**8 (2.4–26.9)**	**23.8 (10.4–54.2)**	**17.6 (6.5–47.6)**	**4.3 (2.1–8.6)**	**20.7 (7.7–55.7)**	**16.4 (5–53.6)**	**8.7 (2.9–26.2)**
**MAL-PDT**	*14.3 (7.1–28.6)*		**2.8 (0.8–9.3)**	**1.1 (0.2–4.9)**	**0.5 (0.1–2.2)**	**1.4 (0.4–5.1)**	**1.1 (0.3–4.1)**	**0.3 (0.1–0.8)**	**1.3 (0.3–4.8)**	**1.0 (0.2–4.4)**	**0.5 (0.1–2.2)**
**ALA-PDT gel**	*40.1 (16.1–100.1)*	*2.7 (1.4–5)*		**0.4 (0.1–2.1)**	**0.2 (0.0–1.0)**	**0.5 (0.1–2.3)**	**0.4 (0.1–1.8)**	**0.1 (0.0–0.4)**	**0.5 (0.1–2.1)**	**0.4 (0.1–1.9)**	**0.2 (0.0–1.0)**
**ALA-PDT patch**	*16.7 (7.3–38.2)*	*-*	*-*		**0.4 (0.1–1.5)**	**1.3 (0.3–5.4)**	**1.0 (0.2–4.1)**	**0.2 (0.1–0.9)**	**1.1 (0.2–5.3)**	**0.9 (0.2–4.8)**	**0.5 (0.1–2.4)**
**Cryotherapy**	*7.3 (2.7–19.4)*	*-*	*-*	*0.5 (0.3–0.8)*		**3.0 (0.7–12.6)**	**2.2 (0.6–8.5)**	**0.5 (0.1–2.1)**	**2.6 (0.5–12.2)**	**2.0 (0.4–11)**	**1.1 (0.2–5.4)**
**Imiquimod 5% (16 week)**	*21.7 (10.9–42.9)*	*-*	*-*	*-*	*-*		**0.7 (0.2–2.6)**	**0.2 (0.1–0.5)**	**0.9 (0.2–3.1)**	**0.7 (0.2–2.9)**	**0.4 (0.1–1.5)**
**Imiquimod 5% (4 week)**	*17.5 (2.4–128.3)*	*-*	*-*	*-*	*2.6 (0.7–10.0)*	*-*		**0.2 (0.1–0.8)**	**1.2 (0.3–4.7)**	**0.9 (0.2–4.3)**	**0.5 (0.1–2.2)**
**Diclofenac 3%**	*3.4 (2.3–4.9)*	*-*	*-*	*-*	*-*	*-*	*-*		**4.8 (1.7–14)**	**3.8 (1–15.3)**	**2.0 (0.5–7.5)**
**5-FU**	*20.5 (4.2–100.5)*	*-*	*-*	*-*	*-*	*-*	*-*	*2.6 (1.7–4.1)*		**0.8 (0.2–3.8)**	**0.4 (0.1–1.9)**
**Ingenol mebutate**	*16.8 (9.2–30.8)*	*-*	*-*	*-*	*-*	*-*	*-*	*-*	*-*		**0.5 (0.1–2.7)**
**Imiquimod 3.75%**	*8.5 (5.1–14.3)*	*-*	*-*	*-*	*-*	*-*	*-*	*-*	-	-	

**Cells in bold font**: Network meta-analysis results (Top row is treatment, Left row is comparator); *Cells in italic font*: Direct meta-analysis results (Left row is treatment, Top row is comparator).

In a sensitivity analysis, ALA-PDT stick was also included in the analyses, resulting in one additional study being added to the NMA [16]. Efficacy of the other treatments were not affected by inclusion of this treatment in the NMA. The highest ranking of treatments according to the SUCRA scores was BF-200 ALA (90.8%), followed by IMI 5% 16-week (71.7%), ALA-PDT stick (69.0%) and 5-FU 0.5% (64.1%). Thus, the efficacy of ALA-PDT stick was ranked below BF-200 ALA and between IMI 5% 16-week and 5-FU 0.5%.

## Discussion

In this study we performed a network meta-analysis to compare the efficacy of available treatments for mild to moderate AK on the face or scalp. The starting point for this analysis was the recent Cochrane review of AK treatments by exploring the relative effect of different PDT treatments, and also different imiquimod preparations. However, we went beyond the Cochrane review [15] and the related NMA publication by Gupta et al [14] to include assessment of the relative efficacies of the separate PDT and imiquimod agents. All treatments showed statistically significant efficacy compared to placebo. BF-200 ALA gel was the most efficacious treatment in terms of complete patient clearance, and also had the highest probability to be the most efficacious treatment among all compared treatments. The SUCRA scores also indicated that BF-200 was the most efficacious treatment. As our study was performed from a European perspective, ALA-PDT stick treatment was not included in our main analysis. However, a scenario analysis showed that this treatment could be the second most effective AK treatment in regions where it is available.

### Strengths of the study

NMAs provide a valid statistical alternative to direct head-to-head studies [10], [11]. An advantage of Bayesian NMAs such as this study over frequentist approaches is the ability to ‘rank’ treatments, either according to probabilities to be the ‘best’ (i.e. most effective) treatment or according to SUCRA scores, which can be useful for clinical treatment decisions and HTA [12], [13], [24]. In a NMA evidence of multiple RCT's can be combined while retaining the randomisation element of these trials. This is opposed to ‘naïve’ meta-analysis, where efficacy data from individual study arms is extracted and pooled as if they were from one large trial. Such pooling approaches may lead to biased efficacy estimates.

The NMA studied the outcome ‘complete patient clearance’. Although other outcome parameters have been reported in various RCT's, such as ‘% reduction in lesion count’, a patient-based measure was preferred because it can be used in health-economic analyses.

The studies considered for inclusion in the NMA were identified in a prior Cochrane review, which used a robust and systematic approach to identify RCT's of interventions for actinic keratosis [15]. Not all studies identified in the Cochrane review were included in our NMA due to our stricter inclusion criteria. For example, we only included studies that evaluated efficacy after a minimum of one month after EOT; this excluded a study that evaluated efficacy immediately at EOT [26]. We also excluded studies with unconventional treatment dosages or schedules, such as 3- or 8-week courses of IMI 5% or a 1-week course of 5-FU [27], because the focus of this study was in comparing and ranking AK treatments that are commonly used in clinical practice in European countries. The efficacy data was extracted from the published manuscripts of the included studies and not from the Cochrane review.

The included studies were similar with regard to average age, gender, number of lesions at baseline and other patient characteristics. Moreover, patient characteristics were not significantly correlated with treatment success. This limits the potential of heterogeneity across trials and consequent bias in the NMA.

### Limitations of the study

The results of this study are subject to several limitations. Although over 5,500 patients were included in the NMA, as in many NMAs the study was limited by the relatively small number of trials. Study covariates were not taken into account in the NMA but were relatively similar across trials; furthermore, there were no significant associations between patient characteristics in the different trials and treatment outcome.

Although a random effects model was used for the NMA, which takes into account study heterogeneity, any differences in trial procedures and settings between the included studies may have influenced results. One area of differences in trial design was the time point of efficacy evaluation. The evaluation time point varied from 4 weeks after EOT (e.g. all IMI 5% 4 week trials), to 8 weeks after EOT (e.g. all IMI 5% 16 week trials) and 12 weeks after EOT (all PDT trials). The influence of these variations in trial design was limited by excluding trials that evaluated efficacy earlier than one month after EOT, because several studies noted a considerable increase in efficacy between the end of treatment visit and post-end of treatment visits [28]–[30].

A limitation of the cryotherapy arm in the NMA was that one of the two included studies used only one treatment session [31], whereas in the other study a second cryotherapy session was allowed although how many patients received a second session was not reported [21].

Concerns have been voiced that differences in placebo and vehicle efficacy might limit the validity of grouping vehicle arms in meta-analyses of AK treatments. The NMA assumes identical placebo efficacy. However, the hyaluronic acid (HA) vehicle used in DHA treatment has previously been discussed with respect to contributing to an enhanced efficacy for placebo [32]. HA enhances the retention and localization of DHA in the epidermis and thereby has a permissive or potentiating effect on AK clearance by DCH [33]. It is unclear whether HA functions only as a drug delivery system or whether HA alone also influences AK lesion clearance. In *in vitro* experiments on colon-26 adenocarcinomas, HA alone appeared to have a small effect on tumour angiogenesis and growth but no effects on cell proliferation or viability [34]. Other studies confirm that the effects of HA alone on tumour angiogenesis are small and unsustained compared to DCH [35]. In animal models, HA alone did not affect vascularity in granulomatous tissue neovasculature (whereas HA with DHA did significantly reduce vascularity) [36], or on cholesteatoma formation in squamous epithelium [37]. Studies on the effectiveness of HA alone on AK lesion clearance however are lacking. Similar arguments may be presented for PDT, where, for the MAL cream and ALA gel studies, lesion preparation in the studies included mild curettage also in the placebo arms, which by itself may have some efficacy [17]–[20]. Therefore, a slight efficacy of the placebo treatment because of curettage may have caused an underestimation of the true effectiveness of the active treatments in these studies.

The absolute clearance as estimated from the NMA generally corresponded to those in the naïve meta-analysis. However, for IMB and IMI 5% (16-week course) the estimated absolute clearance was higher using the NMA compared to the naïve analysis. This was caused by the relatively low placebo response rates in the clinical trials of these drugs [38]–[40]. In general, results of the NMA were accompanied by large variances. Uncertainty in the analysis can be reduced when more studies are performed on the different AK treatments, preferably also including more head-to-head trials of active treatments.

### Discussion of previous studies

Recently, a NMA of treatments in AK based on the Cochrane review was published [14]. This NMA grouped different ALA-PDT treatments, such as ALA applied as a gel, a patch or with a stick. The NMA also grouped different imiquimod treatments and did not include IMI 3.75%. In clinical practice however, these drugs are considered as distinct treatment modalities and are marketed as such [41], [42]. The study also included treatment options that are not available in European clinical practice, such as ALA stick. The published NMA concluded that 5-FU 5.0% was the most efficacious treatment, followed by ALA-PDT. Differences between the NMA from Gupta et al and our NMA may be caused because our study focused on AK on face or scalp and considered different ALA-PDT options as separate treatment modalities as well as different IMI treatments. Due to limitations in data availability our NMA was unable to make a reliable estimation of the efficacy of 5-FU 5.0% and therefore did not include this treatment. Furthermore, our NMA focused on ALA-PDT using narrow-band light sources (LED lights) only. Broad-band light sources have been shown to result in reduced treatment success [19], [20]. Therefore, narrow-band lamps seem to be most relevant for clinical and HTA decision making.

Several previous studies have performed meta-analyses or indirect comparisons of AK treatments. Some of these were narrative [43] or naïve meta-analyses [22] which are open to risk of bias [44]. Two studies performed meta-analyses of placebo-controlled studies of IMI 5% (16-week course) [45], [46]. These studies used fixed-effect models but the results were similar to the random-effects direct meta-analysis in our study. Two studies performed meta-analyses as part of a pharmacoeconomic evaluation [47]–[49]. Two economic evaluations performed naïve meta-analyses [47], [49]. A more recent cost-effectiveness study performed an indirect comparison of IMI 5% (4-week course) and MAL-PDT, using cryotherapy as the common comparator [48]. This comparison however included studies with intra-individual randomization [50] and the treatment outcome ‘complete lesion clearance’ rather than ‘complete patient clearance’ [50], [51]. Our analysis excluded these types of studies.

### Implications for clinicians and policymakers

The results of this study may provide valuable information for the optimal management of AK and for use in HTA and economic evaluations of the cost-effectiveness of alternative AK treatments available in Europe. However, there are some limitations of this NMA for clinical practice and policymaking. Firstly, some treatment options for AK could not be included in the NMA because they could not be linked in the treatment network, such as colchicine and resiquimod. Secondly, in clinical practice some treatment courses may be repeated, such as 5-FU and DFC, but this could not be studied in the NMA as no RCT's studied this. Finally, recurrence of cleared lesions may occur. Observational long-term recurrence data is available for some treatments but these could not be included in a NMA that focuses on RCT evidence as the evidence base for recurrence is limited in RCTs. A recent study has compared recurrences and probabilities for patients to be still cleared after one year for several treatment modalities that are also reviewed here [52]. Our analysis did not take differences in adverse events, cosmetic outcomes and treatment costs into consideration which may also have to be considered in the context of decision-making. However, results from this NMA may provide relative efficacy data to inform future cost-effectiveness studies of AK treatments used in clinical practice in Europe.

## Conclusions

The results from this NMA of available treatments in AK suggest that BF-200 ALA gel, using narrow-band lights, is expected to provide the greatest response in terms of complete patient clearance of AKs on the face and scalp. The NMA ranked BF-200 ALA with the highest probability of being the most efficacious treatment for this outcome measure. This study extends on the recent study of Gupta et al which was also based on the Cochrane review but did not distinguish between alternative PDT agents. Our NMA therefore is relevant for clinical and HTA based decision-making, and can assist in the improved management of AK.

## Supporting Information

Table S1 **Inclusion and exclusion of studies identified in Cochrane review.**
(DOCX)

Checklist S1 **PRISMA Checklist.**
(DOC)
