# Supplementary material for: A Network Meta-Analysis of the Relative Efficacy of Treatments for Actinic Keratosis of the Face or Scalp in Europe
Source: PLoS One. 2014 Jun 3;9(6):e96829. doi: 10.1371/journal.pone.0096829 (PMC4043670; doi:10.1371/journal.pone.0096829)
Supplement: Table S1 — Inclusion and exclusion of studies identified in Cochrane review. (DOCX) [file pone.0096829.s001.docx]

**Table S1: inclusion and exclusion of studies identified in Cochrane review**

| **Author, year *** | **Included?** | **Primary reason for exclusion** | **Comment** |
| --- | --- | --- | --- |
| Akar 2001 |  | No network match | Comparison of two dosages of Colchicine |
| Alberts 2000 |  | No outcome 'complete patient clearance' |  |
| Alirezai 1994 |  | No outcome 'complete patient clearance' |  |
| Alomar 2007 | Included |  |  |
| Anderson 2009 |  | Wrong indication | Non-facial actinic keratoses |
| Bercovitch 1987 |  | No outcome 'complete patient clearance' |  |
| Chen 2003 |  | Non-standard treatment modality | 3-week course of IMI 5% |
| Dragieva 2004a |  | Wrong indication | Immunocompromised participants |
| Fariba 2006 |  | No outcome 'complete patient clearance' |  |
| Foote 2009 |  | No outcome 'complete patient clearance' |  |
| Freeman 2003 |  | No outcome 'complete patient clearance' |  |
| Gebauer 2003 | Included |  |  |
| Gebauer 2009 |  | Wrong indication | More than 20 lesions at baseline |
| Hanke 2010 | Included |  |  |
| Hantash 2006 |  | No outcome 'complete patient clearance' |  |
| Hauschild 2009a | Included |  |  |
| Hauschild 2009b | Included |  |  |
| Hauschild 2009c |  | No network match | Comparison of application times with ALA-PDT patch |
| Huyke 2009 |  | No network match | Cryotherapy compared to betulin-based oleogel |
| Jeffes 2001 |  | Intraindividual study design |  |
| Jorizzo 2002 | Included |  |  |
| Jorizzo 2004 |  | Non-standard treatment modality | 1-week course of 5-FU |
| Jorizzo 2006 |  | Combination therapy | 5-FU followed by Cryotherapy |
| Jorizzo 2007 | Included |  |  |
| Jorizzo 2010 |  | Combination therapy | Cryotherapy following IMI 3.75% |
| Kang 2003 |  | No outcome 'complete patient clearance' |  |
| Kaufman 2008 |  | No outcome 'complete patient clearance' |  |
| Korman 2005 | Included |  |  |
| Kose 2008 |  | No outcome 'complete patient clearance' |  |
| Krawtchenko 2007 | Included |  | 5% 5-FU arm was not included |
| Kulp-Shorten 1993 |  | No outcome 'complete patient clearance' |  |
| Lebwohl 2004 | Included |  |  |
| Loven 2002 |  | Intraindividual study design | Comparison of 5-FU dosage on opposite sides of face |
| McEwan 1997 |  | Wrong indication | Treatment of single lesion |
| Misiewicz 1991 |  | No outcome 'complete patient clearance' |  |
| Moloney 2007 |  | Intraindividual study design | ALA-PDT and MAL-PDT on opposite sides of scalp |
| Moloney 2010 |  | No outcome 'complete patient clearance' |  |
| Moriarty 1982 |  | Intraindividual study design |  |
| Morton 2006 |  | No outcome 'complete patient clearance' |  |
| NCT00774787 |  | Intraindividual study design |  |
| NCT00828568 Aldara | Included |  |  |
| NCT00828568 Taro | Included |  |  |
| Olsen 1991 |  | No outcome 'complete patient clearance' |  |
| Ooi 2006 |  | Timepoint of efficacy evaluation | Evaluation at EOT visit (not at least 1 month after EOT) |
| Ortonne 2010 |  | No outcome 'complete patient clearance' |  |
| Ostertag 2006 |  | No outcome 'complete patient clearance' |  |
| Pariser 2003 |  | Non-standard treatment modality | MAL-PDT with broadband lights |
| Pariser 2008 | Included |  |  |
| Perret 2007 |  | Wrong indication | Immunocompromised participants |
| Persaud 2002 |  | No outcome 'complete patient clearance' |  |
| Photocure-Australia 2004 |  | Non-standard treatment modality | MAL-PDT with broadband lights |
| Photocure-US 2004 |  | Non-standard treatment modality | MAL-PDT with broadband lights |
| Piacquadio 2004 | Included in scenario | Non-standard treatment modality | ALA-PDT stick is not used in EU |
| Rivers 2002 | Included |  |  |
| Seckin 2009 |  | No outcome 'complete patient clearance' |  |
| Shaffelburg 2009 |  | No outcome 'complete patient clearance' |  |
| Siller 2009 |  | No outcome 'complete patient clearance' |  |
| Smith 2003 |  | Non-standard treatment modality | Applied for 1 hour followed by PDT |
| Solaraze study 2 | Included |  |  |
| Sotiriou 2009 |  | No outcome 'complete patient clearance' |  |
| Stockfleth 2002 |  | Non-standard treatment modality | 12-week course of IMI 5% |
| Swanson 2010a | Included |  |  |
| Swanson 2010b |  | Wrong indication | Non-facial actinic keratoses |
| Szeimies 2002 |  | No outcome 'complete patient clearance' |  |
| Szeimies 2004 | Included |  |  |
| Szeimies 2008 |  | No network match | Dose-ranging study of resiquimod |
| Szeimies 2009 | Included |  |  |
| Szeimies 2010b | Included |  |  |
| Tan 2007 |  | Combination therapy | IMI 5% followed by Cryotherapy |
| Tanghetti 2007 |  | Wrong indication | More than 20 lesions at baseline |
| Tarstedt 2005 |  | No network match | Comparison of two modalities of MAL-PDT |
| Thompson 1993 |  | No outcome 'complete patient clearance' |  |
| Tong 1996 |  | No outcome 'complete patient clearance' |  |
| Ulrich 2007 |  | Wrong indication | Immunocompromised participants |
| Ulrich 2010 |  | Wrong indication | Immunocompromised participants |
| Van der Geer 2009 |  | No outcome 'complete patient clearance' |  |
| Von Felbert 2010 |  | No network match | Comparison of two modalities of MAL-PDT |
| Weiss 2002 | Included |  |  |
| Wiegell 2008 |  | No outcome 'complete patient clearance' |  |
| Wiegell 2009 |  | No outcome 'complete patient clearance' |  |
| Wiegell 2011a |  | No outcome 'complete patient clearance' |  |
| Wolf 2001 | Included |  |  |
| Zeichner 2009 |  | No outcome 'complete patient clearance' |  |
| Akarsu 2011 |  | No outcome 'complete patient clearance' |  |
| Apalla 2011 |  | No outcome 'complete patient clearance' |  |
| Azimi 2012 |  | No outcome 'complete patient clearance' |  |
| Damian 2011 |  | No outcome 'complete patient clearance' |  |
| Deonizio 2011 |  | No outcome 'complete patient clearance' |  |
| Dirschka 2012 | Included |  |  |
| Galitzer 2011 |  | Combination therapy | Tazarotene followed by ALA-PDT |
| Haddad 2011 |  | No outcome 'complete patient clearance' |  |
| Lebwohl 2012 | Included |  |  |
| Serra-Guillen 2012 |  | Non-standard treatment modality | MAL-PDT with broadband lights |
| Stockfleth 2011 | Included |  |  |
| Wiegell 2011b |  | Non-standard treatment modality | MAL-PDT with daylight light |

* full references and appraisal of potential bias and study quality can be found in the Cochrane review ^15^
